# *Flavobacterium psychrophilum*: Response of Vaccinated Large Rainbow Trout to Different Strains

**DOI:** 10.3390/biology11121701

**Published:** 2022-11-24

**Authors:** Moonika H. Marana, Inger Dalsgaard, Per Walter Kania, Abdu Mohamed, Jens Hannibal, Kurt Buchmann

**Affiliations:** 1 Department of Veterinary and Animal Sciences, Faculty of Health and Medical Sciences, University of Copenhagen, DK-1870 Frederiksberg, Denmark; 2 Unit for Fish and Shellfish Diseases, National Institute of Aquatic Resources, Technical University of Denmark, DK-2800 Lyngby, Denmark; 3 Department of Clinical Chemistry, Bispebjerg Hospital, DK-2400 Copenhagen, Denmark

**Keywords:** *Flavobacterium psychrophilum*, rainbow trout fry syndrome, bacterial cold water disease, aquaculture, injection vaccination

## Abstract

**Simple Summary:**

Although *Flavobacterium psychrophilum* is recognized as the causative pathogen of rainbow trout fry syndrome (RTFS), often resulting in high fry mortality, it is also responsible for bacterial cold water disease (BCWD) in large and older rainbow trout (*Oncorhynchus mykiss*). These older fish do not experience high mortality, but sustain, through the shedding of bacteria, a constant infection pressure at farm level, which exposes fry to an unnecessary infection risk. We have produced and assessed the immunogenicity of an experimental injection BCWD vaccine, which may be used to decrease the shedding of bacteria from older fish. Significantly elevated antibody titers were found against all serotypes in vaccinated fish. The study suggests that an injection vaccine containing formalin-inactivated whole cells of *F. psychrophilum* (serotype Fd), adjuvanted with FIA, may also induce protection against heterologous strains. We hypothesize that the vaccination of older rainbow trout will reduce the infection pressure in farms and minimize *F. psychrophilum* infection in fry at farm level.

**Abstract:**

Background: Although *Flavobacterium psychrophilum* is recognized as the causative pathogen of rainbow trout fry syndrome (RTFS), often resulting in high fry mortality, it is also responsible for bacterial cold water disease (BCWD) in large and older rainbow trout (*Oncorhynchus mykiss*). These older fish do not experience high mortality, but sustain, through the shedding of bacteria, a constant infection pressure at farm level, which exposes fry to an unnecessary infection risk. We have produced and assessed the immunogenicity of an experimental injection BCWD vaccine, which may be used to decrease the shedding of bacteria from older fish. Methods: A total of 800 fish were i.p.-injected: 200 fish received the bacterin with adjuvant, 200 fish received the bacterin alone, 200 fish received adjuvant alone and 200 fish were injected with physiological saline. Blood samples were taken at day 0 and at three different time points (4, 8 and 14 weeks) post-vaccination. Plasma antibody levels were measured by ELISA for reactivity against both the homologous *F. psychrophilum* vaccine strain (serotype Fd) and heterologous strains (serotype Th). Results: Significantly elevated antibody titers were found against all serotypes in vaccinated fish. Welfare parameters associated with the vaccination process were evaluated by analyzing trout plasma samples for six different biochemical parameters, but no adverse effects associated with injection were indicated. Conclusions: The study suggests that an injection vaccine containing formalin-inactivated whole cells of *F. psychrophilum* (serotype Fd), adjuvanted with FIA, may also induce protection against heterologous strains. We advocate for, as the next step, the performance of field trials evaluating if the vaccination of older rainbow trout will (1) reduce the infection pressure in farms, (2) elevate the general health level in all groups and (3) minimize *F. psychrophilum* infection in fry at farm level. This may reduce the need for the administration of antibiotics in all age classes.

## 1. Introduction

*Flavobacterium psychrophilum* is a psychrophilic Gram-negative bacterium that causes bacterial cold water disease (BCWD) in juvenile and adult rainbow trout [1]. In small fry, the disease is known as rainbow trout fry syndrome (RTFS) [2]. Both disease forms can cause significant economic losses and necessitate frequent antibiotic treatments [3,4,5]. The mortality among older fish is generally lower; disease signs may occur, but surviving fish may act as reservoirs, allowing both horizontal and vertical transmission to other age classes [6,7,8]. Carriers without clinical signs allow *F. psychrophilum* to survive in spleen phagocytic cells [9,10], gonads [8] and eggs [6]. The bacterium is able to survive outside a host for extended periods [11] and benefits from low temperatures, as its optimal temperature range is 10–14 °C. The disease may be triggered by low water quality and poor management strategies, factors causing stress and increased susceptibility to pathogens in general [12]. The main infection control is based on improved management and treatment with antibiotics, but the latter strategy raises concerns about the development of antibiotic resistance in *F. psychrophilum* [13]. In addition, stringent rules apply for organic trout farms, restricting the use of antibiotics to control disease outbreaks. Thus, if the concerned aquaculture animals are to be sold as organic products, the treatments are limited to two courses per year, and only one treatment is accepted if a production cycle is less than a year [14]. Vaccination is generally considered as an effective and protective measure against bacterial infections in fish [15], but no commercial vaccine against *F. psychrophilum* is currently available in Danish aquaculture. The focus on RTFS and vaccine development for fry has diminished efforts for the development of an effective vaccine for older fish. The immature adaptive immune system of small fry only allows a weak response to vaccines [16], and the genetic and serologic variability of *F. psychrophilum* strains further suggests that a range of strains should be included in a vaccine in order to confer protection [17]. We hypothesized that a highly immunogenic vaccine for older and immunocompetent fish may be a way towards broad immunoprotection against the disease. Thus, by immunizing larger and older rainbow trout, the shedding of bacteria from the older fish will decrease, and thereby the infection level in the farm will fall. This will not only benefit the vaccinated older fish, but to a much higher degree protect the vulnerable fry from exposure. We have developed an experimental injection vaccine based on formalin-killed *F. psychrophilum* and vaccinated large rainbow trout. We then evaluated the antibody response in the fish, not only against the vaccine strain, but also against two heterologous strains. We present evidence that the intraperitoneal (i.p.) vaccination of large rainbow trout with fully developed immune systems elicits strong antibody production with broad coverage against homologous and heterologous *F. psychrophilum* strains. The study suggests vaccinating the older and larger trout in the farms. This may lower the general infection pressure and antibiotic usage in all age classes, but the health-improving impact may benefit fry in particular. 

## 2. Materials and Methods

All materials and protocols will be readily available for readers.

### 2.1. Fish

Certified virus-free and disinfected eyed rainbow trout eggs from Hallesø hatchery (Aquasearch Ova, Jutland, Denmark) were transported to the thermostat-controlled Aqua Baltic pathogen-free hatchery [18] (Nexø, Denmark) and hatched at 7 °C across 14 days. Fish were reared in 1 m^3^ fibreglass tanks, each containing 700 L recirculating municipal water (12 °C), and fed 1% biomass of dry pelleted feed (INICIO 917, BioMar A/S, Brande, Denmark) daily. Larger fish, but not fry, were used for vaccination when they reached a uniform body weight of 42 g (±3 g), because we wished to use fish with a fully developed adaptive response. For body weight determination, the fish were weighed together, immediately after fine grading (3 g accuracy). Thereafter, the mean weight was calculated and the fish were randomly allocated to the different fish tanks. 

### 2.2. Vaccine Preparation

For the production of bacterin and the adjuvanted vaccine, we prepared a solution of formalin-inactivated whole cells of *F. psychrophilum* strain 950106-1/1, serotype Fd (1 × 10^9^ CFU/mL), from which preparations with or without Freund’s Incomplete Adjuvant (FIA) (F5506, Sigma-Aldrich, Denmark) were established. The bacterial strain was isolated from a disease outbreak with significant fry mortality in a Danish freshwater rainbow trout farm in 1995 [9]. Swabs were taken from diseased fry, whereafter the isolate was inoculated on tryptone yeast extract (TYE) agar and identified by standard biochemical tests and PCR [9]. For the preparation of the adjuvanted vaccine (termed vaccine in this study), 15 mL of bacterin was combined with 11 mL FIA, and emulsified by vigorous vortexing at room temperature for 1 h. The final cell concentration of the bacterin in the vaccine was 5.8 × 10^8^ CFU/mL. The study also included an adjuvant only inoculum (termed adjuvant in this study), which was prepared by emulsifying 15 mL saline and 11 mL FIA with a bacterin-only inoculum (termed bacterin in this study), which was prepared by combining 15 mL bacterin (1 × 10^9^ CFU/mL) with 11 mL saline. Finally, a 26 mL inoculum of physiological saline (termed saline in this study) was prepared. 

### 2.3. Vaccination

A total of 800 rainbow trout (average body weight of 42 g) were randomly divided into 4 groups of 200 fish, and each group was then further subdivided into duplicates of 100 fish each, which were allocated into 700 L tanks. In brief: we used duplicate tanks, each with 100 fish, for (1) saline injection, (2) adjuvant injection, (3) bacterin injection and (4) vaccine (adjuvanted) injection. The adjuvant used was Freund’s incomplete adjuvant (FIA). The fish were anaesthetized with 100 mg/L MS222 (A5040, Sigma-Aldrich, Denmark) and i.p.-injected with 0.1 mL of the different injection formulations, adjusted to the ambient temperature (12 °C), prior to immunization.

### 2.4. Sampling 

**Figure 1:**
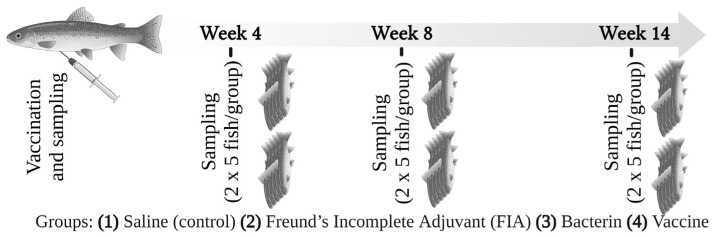
Experimental design and timeline of the vaccination trial and sampling. A total of 10 (2 × 5) fish (n = 10) were sampled from each group at each sampling time point.

Blood samples were taken from 10 control fish prior to vaccination at day 0 and from 10 fish per group (2 × 5 fish in duplicate tanks) at three different time points. In order to follow the development of the adaptive response from early to full development over months, we sampled fish at 4, 8 and 14 weeks post-vaccination (wpv) (Figure 1). Blood samples were collected by caudal vein puncture from randomly netted and euthanized fish (300 mg/L MS222). Blood was collected by heparinized syringes (1 mL volume equipped with BD microlance 25 G 1”no. 18, Becton Dickinson S.A. Madrid, Spain) and transferred into Eppendorf tubes, whereafter plasma was separated at 4 °C by centrifugation (3000× *g*) for 10 min and stored at −80 °C until further analysis.

### 2.5. Enzyme-Linked Immunosorbent Assay (ELISA) for Detection of Ig in Plasma

Antibody reactivity was measured by ELISA according to our previous study [19]. In brief, 96-well microtiter plates (MaxiSorp™ Nunc, Thermo Fisher Scientific, Soborg, Denmark) were coated with 5 µg/mL sonicated bacterial lysate. All 130 plasma samples were tested against the vaccine strain 950106-1/1 (serotype Fd). In addition, we also compared plasma antibody reactivity in fish from the vaccine group (30 samples) and the corresponding control fish to different strains and serotypes of the bacterium (strains 950106-1/1 (serotype Fd)), 180524-1/1 (serotype Th) and 151127-1/7H (serotype Th)). Serotyping was conducted by multiplex PCR according to [20]. Based on pilot measurements, a 1:1000 plasma dilution was chosen. The diluent used was 0.1% Bovine Serum Albumin (BSA, A4503, Sigma-Aldrich, Soborg, Denmark) in washing buffer. A plasma sample (100 µL) was added to each duplicate well and incubated at 4 °C overnight. After plate washing, a volume of 100 µL mouse anti-salmonid Ig (MCA2182 Bio-Rad, Denmark, diluted 1:500, 1 h) was added. Finally, 100 µL HRP-conjugated rabbit anti-mouse IgG (STAR13B, Bio-Rad, Denmark diluted 1:500, 1 h) was applied, before the color reaction was developed with 100 µL tetramethylbenzidine (TMB) PLUS substrate (BUF042A, Bio-Rad, Soborg, Denmark) and stopped after 4 min (100 µL 1N HCl per well). To determine the non-specific background binding of antibodies to solid surfaces of the microtiter plate wells, all samples were also tested in non-coated wells. The optical density (O.D.) values were subtracted from those obtained with antigen-coated wells. All the samples were normalized according to the plate-specific regulation factor, i.e., the ratio of the mean absorbance from the entire pool of control wells to the mean absorbance of the positive control wells on each plate. The O.D. was measured at 450 nm in an Epoch spectrophotometer (BioTek, Winooski, VT, USA) in duplicate wells.

### 2.6. Blood Parameters

Selected blood parameters were analyzed for all blood samples in duplicate on the Cobas 8000 (Roche Diagnostics, Mannheim, Germany) by absorption photometry. Plasma samples were analyzed for 6 different biochemical parameters. Methods are described (www.roche.com accessed on 10 January 2021) with reference and system codes: alanine aminotransferase (ALAT) [21] (ACN 8681), alkaline phosphatase (ALP) [22] (ACN 8683), cholesterol (CHOL) [23] (ACN 8798), high-density lipoprotein cholesterol (HDL) [24] (ACN 8454), total protein (TP) [25] (ACN 8679) and triglycerides (TRIG) [26] (ACN 8781). 

### 2.7. Data Analysis

All statistical tests were performed using GraphPad Prism version 9 (GraphPad Software, USA) and *p*-values < 0.05 were considered statistically significant. Normality was examined by the Kolmogorov–Smirnov test. One ELISA titer group did not pass the test and, therefore, ELISA O.D. values were compared using the non-parametric Kruskal–Wallis test with Dunn’s multiple comparison test. Blood parameters all passed the normality test and were analyzed with the Student’s *t*-test.

### 2.8. Ethics

All experiments were performed according to the EU Directive 2010/63/EU for experimental animals and the ethical guidelines of the University of Copenhagen. The trial was performed under the license no. 2019-15-0201-00388 under the Experimental Animal Inspectorate, Committee for Experimental Animals, Ministry of Environment and Food, Denmark.

## 3. Results

### 3.1. ELISA

#### 3.1.1. Antibody Reactivity against *F. psychrophilum* Strain 950106-1/1

**Figure 2:**
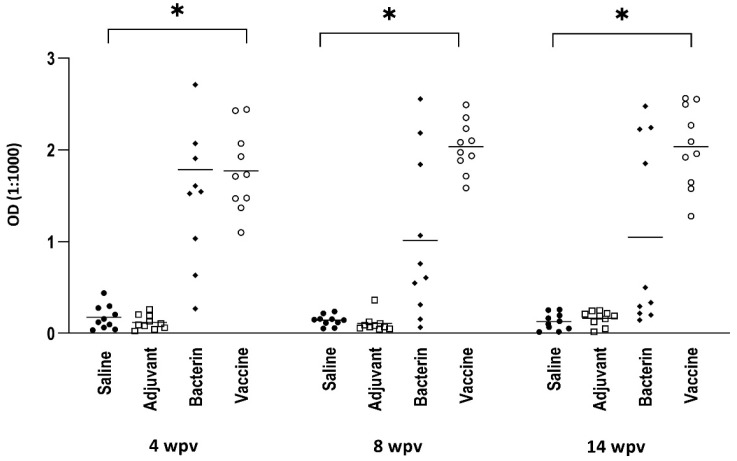
Trout plasma antibody reactivity against the vaccine strain 950106-1/1. ELISA was performed using plasma (1:1000) from 4 different fish groups and measured at 4, 8 and 14 weeks post-vaccination (wpv). O.D. values were compared using Kruskal–Wallis test with Dunn’s multiple comparison test. *: *p* < 0.05. A total of 10 (2 × 5) fish were sampled at each time point from each group. One extreme outlier in the bacterin group at 4 wpv was removed.

Plasma immunoglobulin reactivity to the *F. psychrophilum* vaccine strain was highest in the group of fish injected with the vaccine (adjuvanted bacterin) (Figure 2). Significantly higher reactions in this group, when compared to the control group, were recorded at all three sampling time points. Injection of the bacterin (without adjuvant) resulted in elevated, but non-significant, immunoglobulin levels that decreased over time. The adjuvant alone did not induce any antibody response.

#### 3.1.2. Antibody Cross-Reactivity against Heterologous *F. psychrophilum* Strains 

**Figure 3:**
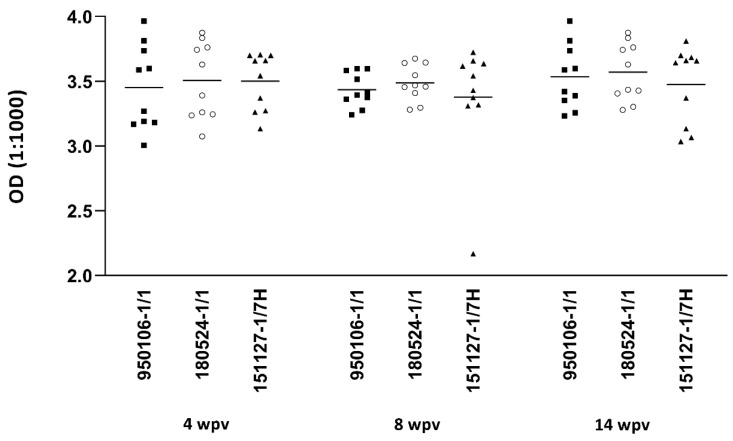
Trout plasma antibody cross-reactivity. ELISA was performed using plasma (1:1000) of vaccinated fish (vaccinated with formalin-killed whole cells of *F. psychrophilum* strain 950106-1/1 adjuvanted with Freund’s Incomplete Adjuvant) and measured at 4, 8 and 14 weeks post-vaccination (wpv). Reactivity was measured towards three different *F. psychrophilum* strains: 950106-1/1 (serotype Fd), 180524-1/1 (serotype Th) and 151127-1/7H (serotype Th). O.D. values were compared using Kruskal–Wallis test with Dunn’s multiple comparison test. *p* < 0.05. Reactions did not differ between serotypes, indicating high cross-reaction between strains.

When the plasma from the vaccine-injected fish was tested for cross-reactivity to all three strains of *F. psychrophilum*, the plasma antibodies reacted to the same extent with the two heterologous strains of *F. psychrophilum* (180524-1/1 and 151127-1/7H, both serotype Th), as was found with the vaccine strain (950106-1/1, serotype Fd). The reactivity was stable over the entire study period (Figure 3). Control fish plasma did not react significantly to any of the isolates (data not shown).

### 3.2. Blood Parameters

The plasma activity of alanine transaminase (ALAT) was significantly elevated in the groups injected with adjuvant only and bacterin only at 4 wpv and in a group injected with the adjuvanted vaccine at 14 wpv (Table 1). The cholesterol level in the group injected with the vaccine was significantly lower when compared to the control group at 4 wpv. Significantly lower high-density lipoprotein cholesterol (HDL) levels were registered in all the groups when compared to the control group (saline injected) at 4 wpv. No significant changes were recorded for the other measured parameters (ALP, TP and TRIG).

## 4. Discussion

The immune system of fish provides a good basis for the development of vaccines, which has resulted in the application of vaccination strategies for a range of aquaculture fish species [15]. We have shown in the present study that older and larger rainbow trout respond very well to an adjuvanted *F. psychrophilum* injection vaccine. We used duplicate groups throughout the study, although triplicate approaches could strengthen the results. No mortality among experimental fish occurred during the experiment, and we demonstrated that broadly reacting antibodies (targeting various serotypes) persisted in the blood of vaccinated fish throughout the 14-week study period. This suggests that larger rainbow trout in an organic farm setting (with limited access to antibiotic treatment) may benefit from vaccination. This reduces, first of all, the need for antibiotic treatment and elevates general health and welfare. In addition, due to a lowered bacterial shedding from the older carrier fish (if they are vaccinated), the general bacterial concentration at farm level may be reduced, which will benefit other age groups, including fry, through herd immunity. Thus, if older fish are protected by vaccination, the youngest age classes of rainbow trout (0.2–2 g fry), which generally are highly susceptible to RTFS caused by *F. psychrophilum,* may be less exposed to the disease in farms where production water is shared between age classes. No effective vaccine is available for these very young fish because the maturation of adaptive immunity in the fry stage is incomplete and immunization is consequently less successful. A pilot study on the immersion vaccination of somewhat older (5 g) rainbow trout fry conferred some protection to the fish when they were challenged at a size of 12 g [17], but experiments have not been able to document the protection of the smallest and most vulnerable fry stage [27]. Immersion vaccination is considered less effective than injection vaccination [28], but the fry stage of rainbow trout is not suitable for injection vaccination. An injection vaccine for large stages of Atlantic salmon is available in Chile, but no corresponding vaccine is at present available for rainbow trout in Europe. Our approach is therefore to target a vaccine for older fish. This will benefit them directly and reduce the need for treatment. As an additional result, the bacterial spread from older fish to younger generations is likely to decrease, and thereby the general *F. psychrophilum* infection pressure in the system may decrease. We documented the broad reactivity (IgM) of vaccinate plasma towards several bacterial strains, which may solve problems with the well-known diversity of *F. psychrophilum* strains [29]. Bacterial heterogeneity has been considered an additional challenge to the development of an effective vaccine for fry. The evidence presented here suggests, nonetheless, that only one serotype of *F. psychrophilum* will be sufficiently immunogenic, and broadly protective, when applied in an adjuvanted vaccine for immune-competent fish. The adjuvant used in this study is a water-in-oil emulsion, a strong inducer of non-specific immune responses and antibody production, which explains the high immunogenicity. Antibodies have previously been suggested to contribute to protection against *F. psychrophilum* [17,30,31,32], although innate responses were also considered protective [33]. 

These preliminary results and their implications for protection and cross-protection should be validated by infection trials using challenges with different strains of *F. psychrophilum.* Likewise, the hypothesized induction of herd immunity should be investigated. The next controlled validation step should measure if the *F. psychrophlum* concentration in the water environment of exposed but vaccinated fish is lower compared to water with non-vaccinated fish. The cross-reactivity of antibodies to the three different *F. psychrophilum* strains suggests that different strains have the antigenic similarities previously noted [17,32,34,35,36,37]. If confirmed, this implies that an adjuvanted monovalent injection vaccine would be a sufficient tool for future vaccination approaches at farm level, even if the pathogen strains differ between farms. Such a strategy would also lower industrial expenses connected to vaccine production.

The blood biochemistry of rainbow trout is influenced by various factors [38]. Stress, disease, nutrition and environmental aspects can cause significant changes in fish blood parameters [39,40,41,42], which suggests that fish blood biochemical parameters may be applied as physiological state indicators [43]. As the application of adjuvants in fish vaccines has raised concerns for fish welfare [44], we addressed this question by investigating the general health profile (reflected by a series of blood parameters) of our experimental fish exposed to vaccination. The significantly higher level of alanine transaminase (ALAT) in some groups, in particular 14 weeks after vaccination with the adjuvanted injection vaccine, when compared to the control group, may indicate a stress effect on the liver, probably in connection to elevated innate responses. Nonetheless, it is noteworthy that the levels of ALAT found in our study were considerably lower when compared to the normal ranges found in rainbow trout [45]. Therefore, caution should be taken when interpreting and comparing these values between different studies, as several factors (temperature, sampling and analytical techniques, fish size, strain, physical condition) may influence the result [45]. Significantly lower cholesterol levels at 4 wpv were found in a group injected with the vaccine. In addition, a lower level of high-density lipids (HDL) was found in all the experimental groups, when compared to the control group, which could indicate changes in cholesterol metabolism and lower feeding activity in these groups shortly after injection. However, no long-term change was indicated in these parameters as the levels of both CHOL and HDL were similar to the control group at later sampling time points. 

## 5. Conclusions

The development of an effective vaccine against BCWD may contribute to the control of the disease and a reduction in antibiotics in the fish farm industry. This is especially important for organic trout farms due to legislation-based restricted access to antibiotic treatments, whereby older fish, through vaccination, may obtain a higher health status in these farms. The present study also suggests that an adjuvanted vaccine for older fish may minimize the infection pressure in farms and secure herd immunity. The lowered infection pressure may particularly benefit the youngest and most vulnerable stages, which cannot be effectively vaccinated against RTFS due to the immature immune system in fry. Furthermore, the observed broad antibody reactivity induced by the formalin-killed *F. psychrophilum* bacterin, based on one bacterial serotype only, but formulated with a FIA adjuvant, suggests that vaccine production expenses can be reduced, because one vaccine strain is sufficient. The stable plasma parameters indicated that the vaccine did not induce severe physiological disturbances. However, challenge studies must be the next step to evaluate the hypothesized vaccine-induced protection. These should target a reduced shedding of *F. psychrophilum* from older vaccinated fish to the fish tank water, and compare these levels to fish tanks with non-vaccinates.

## Figures and Tables

**Table 1:** Plasma parameters in different groups of fish. The blood parameters were analyzed in duplicate by absorption photometry using a Cobas 8000 apparatus (Roche Diagnostics, Denmark). Groups were compared to the saline control group using Student’s *t*-test, *: *p* < 0.05. A total of 10 (2 × 5) (n = 10) fish were sampled at each time point from each group.

	ALAT U/L	ALP U/L	CHOL mmol/L	HDL mmol	TP g/L	TRIG mmol
	Mean ± SEM	Mean ± SEM	Mean ± SEM	Mean ± SEM	Mean ± SEM	Mean ± SEM
4 wpv						
Saline	1.18 ± 0.3	74.01 ± 6.74	6.76 ± 0.35	1.68 ± 0.08	27.28 ± 0.95	2.26 ± 0.35
Adjuvant	2.36 ± 0.38 *	53.37 ± 6.62	5.78 ± 0.41	1.28 ± 0.10 *	24.48 ± 1.25	2.60 ± 0.32
Bacterin	2.46 ± 2.68 *	81.97 ± 21.07	5.64 ± 0.64	1.31 ± 0.1 *	24.66 ± 1.35	2.38 ± 0.24
Vaccine	1.46 ± 0.32	64.66 ± 9.16	5.29 ± 0.25 *	1.25 ± 0.09 *	27.36 ± 1.49	2.52 ± 0.28
8 wpv						
Saline	4.69 ± 0.83	101.84 ± 10.58	8.33 ± 0.49	1.57 ± 0.06	34.52 ± 1.6	3.51 ± 0.33
Adjuvant	4.02 ± 0.67	90.4 ± 10.37	7.62 ± 0.44	1.54 ± 0.04	34.54 ± 1.17	3.18 ± 0.37
Bacterin	3.49 ± 0.52	81.76 ± 9.05	7.14 ± 0.47	1.52 ± 0.06	32.5 ± 1.53	2.73 ± 0.25
Vaccine	4.4 ± 0.43	81.91 ± 7.57	3.39 ± 0.28	1.52 ± 0.03	35.18 ± 1	3.39 ± 0.35
14 wpv						
Saline	5.34 ± 0.85	77.38 ± 11.25	8.55 ± 0.72	1.64 ± 0.08	30.78 ± 1.9	3.15 ± 0.53
Adjuvant	3.51 ± 0.40	66.05 ± 9.41	7.42 ± 0.71	1.66 ± 0.01	28.42 ± 2.22	2.54 ± 0.26
Bacterin	8.37 ± 1.42	89.95 ± 11.71	8.25 ± 0.29	1.79 ± 0.05	31.99 ± 0.82	2.73 ± 0.1
Vaccine	10.76 ± 1.93 *	108.92 ± 16.74	9.11 ± 0.51	1.69 ± 0.05	35.02 ± 1.33	3.88 ± 0.57

## Data Availability

All materials and protocols will be readily available for the readers.

## References

[1] Borg A.F. (1948). Studies on Myxobacteria Associated with Diseases in Salmonid Fishes. Ph.D. Thesis.

[2] Lorenzen E., Dalsgaard I., From J., Hansen E.M., Hørlyck V., Korsholm H., Mellergaard S., Olesen N.J. (1991). Preliminary investigations of fry mortality syndrome in rainbow trout. Bull. Eur. Assoc. Fish Pathol..

[3] Antaya C. (2008). Current eco-economical impacts of *Flavobacterium psychrophilum*. MMG 445 Basic Biotechnol. J..

[4] Lorenzen E., Dalsgaard I., Bernardet J.-F. (1997). Characterization of isolates of *Flavobacterium psychrophilum* associated with coldwater disease or rainbow trout fry syndrome I: Phenotypic and genomic studies. Dis. Aquat. Org..

[5] Starliper C.E. (2011). Bacterial coldwater disease of fishes caused *by Flavobacterium psychrophilum*. J. Adv. Res..

[6] Kumagai A., Yamaoka S., Takahashi K., Fukuda H., Wakabayashi H. (2000). Waterborne Transmission of *Flavobacterium psychrophilum* in Coho Salmon Egg. Fish Pathol..

[7] Madetoja J., Nyman P., Wiklund T. (2000). *Flavobacterium psychrophilum*, invasion into and shedding by rainbow trout *Oncorhynchus mykiss*. Dis. Aquat. Org..

[8] Madsen L., Møller J.D., Dalsgaard I. (2005). *Flavobacterium psychrophilum* in rainbow trout, *Oncorhynchus mykiss* (Walbaum), hatcheries: Studies on broodstock, eggs, fry and environment. J. Fish Dis..

[9] Dalsgaard I., Madsen L. (2000). Bacterial pathogens in rainbow trout, *Oncorhynchus mykiss* (Walbaum), reared at Danish freshwater farms. J. Fish Dis..

[10] Evensen Ø., Lorenzen E. (1996). An immunohistochemical study of *Flexibacter psychrophilus* infection in experimentally and naturally infected rainbow trout (*Oncorhynchus mykiss*) fry. Dis. Aquat. Org..

[11] Madetoja J., Nystedt S., Wiklund T. (2003). Survival and virulence of *Flavobacterium psychrophilum* in water microcosms. FEMS Microbiol. Ecol..

[12] Nematollahi A., Decostere A., Pasmans F., Haesebrouck F. (2003). *Flavobacterium psychrophilum* infections in salmonid fish. J. Fish Dis..

[13] Bruun M.S., Schmidt A.S., Madsen L., Dalsgaard I. (2000). Antimicrobial resistance patterns in Danish isolates of *Flavobacterium psychrophilum*. Aquaculture.

[14] The Council of the European Union (2007). On Organic Production and Labelling of Organic Products and Repealing Regulation (EEC) No 2092/91.

[15] Brudeseth B.E., Wiulsrød R., Fredriksen B.N., Lindmo K., Løkling K.-E., Bordevik M., Steine N., Klevan A., Gravningen K. (2013). Status and future perspectives of vaccines for industrialised fin-fish farming. Fish Shellfish Immunol..

[16] Buchmann K., Buchmann K., Secombes C.J. (2022). Development of Immunocompetence in Fish. Principles in Fish Immunology.

[17] Hoare R., Ngo T.P.H., Bartie K.L., Adams A. (2017). Efficacy of a polyvalent immersion vaccine against *Flavobacterium psychrophilum* and evaluation of immune response to vaccination in rainbow trout fry (*Onchorynchus mykiss* L.). Vet. Res..

[18] Xueqin J., Kania P.W., Buchmann K. (2012). Comparative effects of four feed types on white spot disease susceptibility and skin immune parameters in rainbow trout, *Oncorhynchus mykiss* (Walbaum). J. Fish Dis..

[19] Marana M.H., Sepúlveda D., Chen D., Al-Jubury A., Jaafar R.M., Kania P.W., Henriksen N.H., Krossøy B., Dalsgaard I., Lorenzen N. (2019). A pentavalent vaccine for rainbow trout in Danish aquaculture. Fish Shellfish Immunol..

[20] Rochat T., Fujiwara-Nagata E., Calvez S., Dalsgaard I., Madsen L., Calteau A., Lunazzi A., Nicolas P., Wiklund T., Bernardet J.-F. (2017). Genomic Characterization of *Flavobacterium psychrophilum* Serotypes and Development of a Multiplex PCR-Based Serotyping Scheme. Front. Microbiol..

[21] Bergmeyer H.U., Hørder M., Rej R. (1986). International Federation of Clinical Chemistry (IFCC) Scientific Committee, Analytical Section: Approved recommendation (1985) on IFCC methods for the measurement of catalytic concentration of enzymes. Part 3. IFCC method for alanine aminotransferase (L-alanine: 2-oxoglutarate aminotransferase, EC 2.6.1.2). J. Clin. Chem. Clin. Biochem..

[22] King E.J., Armstrong A.R. (1934). A convenient method for determining serum and bile phosphatase activity. Can. Med. Assoc. J..

[23] Allain C.C., Poon L.S., Chan C.S.G., Richmond W., Fu P.C. (1974). Enzymatic Determination of Total Serum Cholesterol. Clin. Chem..

[24] Kimberly M.M., Leary E.T., Cole T.G., Waymack P.P. (1999). Selection, Validation, Standardization, and Performance of a Designated Comparison Method for HDL-Cholesterol for Use in the Cholesterol Reference Method Laboratory Network. Clin. Chem..

[25] Weichselbaum T.E. (1946). An accurate and rapid method for the determination of proteins in small amounts of blood serum and plasma. Am. J. Clin. Pathol..

[26] Bucolo G., David H. (1973). Quantitative Determination of Serum Triglycerides by the Use of Enzymes. Clin. Chem..

[27] Henriksen N., Lorenzen N., Dalsgaard I., Clausen T., Buchmann K. (2017). Autovaccine-Forsøg (YDS) på Dansk Dambrug. www.danskakvakultur.dk.

[28] Chettri J.K., Deshmukh S., Holten-Andersen L., Jafaar R.M., Dalsgaard I., Buchmann K. (2013). Comparative evaluation of administration methods for a vaccine protecting rainbow trout against *Yersinia ruckeri* O1 biotype 2 infections. Vet. Immunol. Immunopathol..

[29] Madsen L., Dalsgaard I. (2000). Comparative studies of Danish *Flavobacterium psychrophilum* isolates: Ribotypes, plasmid profiles, serotypes and virulence. J. Fish Dis..

[30] LaFrentz B.R., LaPatra S.E., Jones G.R., Cain K.D. (2003). Passive immunization of rainbow trout, *Oncorhynchus mykiss* (Walbaum), against *Flavobacterium psychrophilum*, the causative agent of bacterial coldwater disease and rainbow trout fry syndrome. J. Fish Dis..

[31] Fredriksen B.N., Olsen R.H., Furevik A., Souhoka R.A., Gauthier D., Brudeseth B. (2013). Efficacy of a divalent and a multivalent water-in-oil formulated vaccine against a highly virulent strain of *Flavobacterium psychrophilum* after intramuscular challenge of rainbow trout (*Oncorhynchus mykiss*). Vaccine.

[32] Madetoja J., Lönnström L.G., Björkblom C., Uluköy G., Bylund G., Syvertsen C., Gravningen K., Norderhus E.A., Wiklund T. (2006). Efficacy of injection vaccines against *Flavobacterium psychrophilum* in rainbow trout, *Oncorhynchus mykiss* (Walbaum). J. Fish Dis..

[33] Wiklund T., Dalsgaard I. (2002). Survival of *Flavobacterium psychrophilum* in rainbow trout (*Oncorhynchus mykiss*) serum in vitro. Fish Shellfish Immunol..

[34] Holt R.A. (1987). *Cytophaga psychrophila*, the Causative Agent of Bacterial Cold-Water Disease in Salmonid Fish. Ph.D. Thesis.

[35] Lorenzen E. (1994). Studies on *Flexibacter psychrophilus* in Relation to Rainbow Trout Fry Syndrome (RTFS). Ph.D. Thesis.

[36] Hoare R., Jung S.J., Ngo T.P.H., Bartie K., Bailey J., Thompson K.D., Adams A. (2019). Efficacy and safety of a non-mineral oil adjuvanted injectable vaccine for the protection of Atlantic salmon (*Salmo salar* L.) against *Flavobacterium psychrophilum*. Fish Shellfish Immunol..

[37] Ma J., Bruce T.J., Sudheesh P.S., Knupp C., Loch T.P., Faisal M., Cain K.D. (2019). Assessment of cross-protection to heterologous strains of *Flavobacterium psychrophilum* following vaccination with a live-attenuated coldwater disease immersion vaccine. J. Fish Dis..

[38] Coskun O.F., Aydin D., Duman F. (2016). Comparison of some blood parameters of rainbow trout (*Oncorhynchus mykiss*) living in running and still water. Iran J. Fish Sci..

[39] Chen Y.E., Jin S., Wang G.L. (2005). Study on blood physiological and biochemical indices of *Vibrio alginolyticus* disease of *Lateolabrax japonicus*. J. Oceanogr. Taiwan Strait..

[40] Cnaani A., Tinman S., Avidar Y., Ron M., Hulata G. (2004). Comparative study of biochemical parameters in response to stress in *Oreochromis aureus*, *O. mossambicus* and two strains of *O. niloticus*. Aquac. Res..

[41] Hughes G.M., Nemcsók J. (1988). Effects of low pH alone and combined with copper sulphate on blood parameters of rainbow trout. Environ. Poll..

[42] Peres H., Santos S., Oliva-Teles A. (2014). Blood chemistry profile as indicator of nutritional status in European seabass (*Dicentrarchus labrax*). Fish Physiol. Biochem..

[43] De Pedro N., Guijarro A.I., López-Patiño M.A., Martínez-Álvarez R., Delgado M.J. (2005). Daily and seasonal variations in haematological and blood biochemical parameters in the tench, *Tinca tinca* Linnaeus, 1758. Aquac. Res..

[44] Midtlyng P.J., Reitan L.J., Speilberg L. (1996). Experimental studies on the efficacy and side-effects of intraperitoneal vaccination of Atlantic salmon (*Salmo salar* L.) against furunculosis. Fish Shellfish Immunol..

[45] Manera M., Britti D. (2006). Assessment of blood chemistry normal ranges in rainbow trout. J. Fish Biol..

